# Tension-Type Headache in Early Adolescents: Exploring the Predictive Role of Anxiety and Alexithymia

**DOI:** 10.1192/j.eurpsy.2022.1126

**Published:** 2022-09-01

**Authors:** F. Bizzi, S. Charpentier Mora, A. Riva, M. Tironi, R. Nacinovich

**Affiliations:** 1 University of Genoa, Department Of Educational Sciences (disfor), Genoa, Italy; 2 San Gerardo Hospital, Child Neuropsychiatry Clinic, Monza, Italy

**Keywords:** Tension-Type Headache, Early Adolescents, alexithymia, Anxiety

## Abstract

**Introduction:**

Primary Headache, including Tension-Type Headache (TTH), represents one of the most common somatic disorders in children and adolescents with a strong impact on quality of life. Several risk factors, as environmental, familiar, and psychological features, including personality traits, are related to the development of Primary Headache. However, studies on specific subgroups of TTH are relatively few in early adolescents.

**Objectives:**

Therefore, this cross-sectional pilot study aims at exploring the role of anxiety and alexithymia in early adolescents with and without TTH.

**Methods:**

A sample of 70 early adolescents (M_age_=14.59, SD=1.85; 71% females) consisting of a clinical group (31 with TTH) enrolled in an Italian Child Neuropsychiatry Clinic and a comparison group (38 without TTH) enrolled in schools, matched on gender and age, completed: 1) Multidimensional Anxiety Scale for Children (MASC) to detect the Total levels of Anxiety, also in their factor of Physical Symptoms, Social Anxiety, Harm Avoidance, and Separation Anxiety; 2) the Toronto Alexithymia Scale (TAS-20) to detect the Total levels of Alexithymia, also in their factor of Difficulty to Identifying and to Describing Feelings and Externally Oriented Thinking.

**Results:**

TTH outcome positively correlated with Harm Avoidance (rho=.68, p<.001) and Total Alexithymia (rho=.72, p<.001). In a logistic regression, Harm Avoidance and Total Alexithymia predicted 69% of the variance in TTH outcome (p<.032).

**Conclusions:**

This disorder may be a maladaptive strategy to cope with problems and feeling emotions, then early adolescents could be fostered in the acquisition of more adaptive emotion regulation abilities.

**Disclosure:**

No significant relationships.

